# Systematic review of hearing loss in dental professionals

**DOI:** 10.1093/occmed/kqad084

**Published:** 2023-09-08

**Authors:** J C Hartland, G Tejada, E J Riedel, A H-L Chen, O Mascarenhas, J Kroon

**Affiliations:** School of Medicine and Dentistry, Griffith University, Gold Coast, Queensland 4222, Australia; School of Medicine and Dentistry, Griffith University, Gold Coast, Queensland 4222, Australia; School of Medicine and Dentistry, Griffith University, Gold Coast, Queensland 4222, Australia; School of Medicine and Dentistry, Griffith University, Gold Coast, Queensland 4222, Australia; School of Medicine and Dentistry, Griffith University, Gold Coast, Queensland 4222, Australia; School of Medicine and Dentistry, Griffith University, Gold Coast, Queensland 4222, Australia

## Abstract

**Background:**

Hearing loss leads to increased irritability and disengagement in social activities and conversations, which may impact quality of life. Dental professionals are at risk of developing hearing loss through daily exposure to noise from a wide range of equipment that produces significantly high decibels and noise frequencies.

**Aims:**

The aim of this systematic review was to investigate the risk of hearing loss in dental professionals, including dentists, dental specialists, dental hygienists and dental assistants.

**Methods:**

This review was conducted following the *Cochrane Handbook for Systematic Reviews*. PubMed, Scopus, Embase, Cochrane, Science Direct, Google Scholar and ProQuest were searched up to March 2023. Seventeen of 416 studies met the inclusion criteria. Quality assessment was performed according to the Newcastle–Ottawa Scale for cohort and case–control studies, and a modified version of this tool for cross-sectional studies.

**Results:**

The majority of included studies (82%) found a positive association with hearing loss for dentists and dental specialists, with years of clinical experience identified as a prominent risk factor. Dental hygienists and dental assistants were less commonly reported in the literature. Difference between the left and right ears was found in 71% of studies, with the left ear more affected in both dentists and dental assistants due to proximity to the noise-inducing equipment.

**Conclusions:**

Dental professionals are at risk of hearing loss in their workplace, especially linked to years of clinical experience, which highlights the need for prevention and appropriate ear-protective devices.

## Teaser

A systematic review was conducted to summarize the risk of hearing loss in dental professionals due to exposure to noise-inducing equipment. The results show a clear association with hearing loss in dentists and dental specialists, with years of clinical experience identified as a prominent risk factor. The left ear seems to be more affected due to proximity to the noise-inducing equipment.

**Key learning points**

**What is already known about this subject:**

- Exposure to noise exceeding 85 dB for over 8 h a day may lead to adverse effects on the human ear and can lead to conditions such as noise-induced hearing loss.
- Tinnitus, a condition where an individual experiences a ringing, hissing and sizzling sensation without an external stimulus present, may arise from noise-induced hearing loss.
- Dental professionals are at risk of developing noise-induced hearing loss since they are exposed to a wide range of equipment in their work environment that produces significantly high decibels and frequencies of noise.

**What this study adds:**

- A positive association between noise in the dental workplace and hearing loss was found in dentists and dental specialists, with the left ear more affected, due to proximity to the noise-inducing equipment.
- Results for gender and hours of exposure per day showed no association with hearing loss.
- Future research should investigate the association between noise-induced hearing loss and dental specialists in more detail; the viability of hearing protection targeted towards the ear most at risk; noise levels produced by specific dental equipment; and comparisons between new and old dental equipment.

**What impact this may have on practice or policy:**

- The results from this review highlight the need for prevention and appropriate protective devices to mitigate the implications of noise-induced hearing loss in the dental workplace.
- Digital noise excluding headphones that excludes environmental noise, but allows the passage of the human voice, should be considered for the dental environment.

## Introduction

Noise, measured in decibels (dB), is a stimulus that is perceived through the hearing of the individual and is characterized by the frequency and level of sound [1]. According to the USA’s National Institute on Deafness and Other Communication Disorders (NIDCD), exposure to long or repeated noise exceeding 85 dBA may lead to adverse effects on the human ear [2]. The Occupational Safety and Health Administration (OSHA) further recommends a hearing conservation programme should this exposure exceed an 8-h time-weighted average [3]. The NIDCD describes noise-induced hearing loss (NIHL) as damage to the sensitive structures in the inner ear, caused by both loud and long-lasting sounds [2]. NIHL occurs when sensory cells present in the cochlea are damaged and killed through aggravated noise exposure; they are then unable to regenerate and are eventually replaced by scar tissue, which then results in hearing loss [4]. NIHL should be distinguished from presbycusis, which is age-related hearing loss, usually affecting both ears, due to changes in the middle ear and the nerve pathways from the ear to the brain [5]. Tinnitus, a condition where an individual experiences a constant or intermittent ringing, hissing and sizzling sensation without an external stimulus present, may arise from NIHL [6]. Persistent tinnitus can become chronic, which may require therapy, drugs and hearing aids [7]. Hearing loss leads to increased irritability and disengagement in social activities and conversations, which may be a barrier to the development or management of relationships and affect quality of life [8]. NIHL also leads to an increase in sleep disorders, headaches, heart disease and tinnitus [1].

Dental professionals are at risk of developing NIHL since they are exposed to a wide range of equipment in their work environment that produces significantly high dB and frequencies of noise [9]. Some of these include high-volume excavators, air turbine handpieces, ultrasonic scalers, amalgamators and sterilization equipment [10]. Sound levels of this equipment, often used in combination, were found to vary between 60–99 [1] and 52–92 dBA [9] in studies conducted in two dental schools. A study in four dental practices found that ultrasonic scalers were the only equipment to exceed 85 dBA [10].

The literature reports conflicting results on whether noise in the dental environment results in hearing loss [11]. This systematic review investigated NIHL in dental professionals, including dentists, dental specialists, dental hygienists and dental assistants.

## Methods

This systematic review did not require ethics approval; the protocol was registered on PROSPERO (CRD42022298641). The *Cochrane Handbook for Systematic Reviews* was used to conduct this study [12]. The Preferred Reporting Items for Systematic Reviews and Meta-Analyses (PRISMA) 2020 statement was used for reporting this review [13].

The Population–Exposure–Comparison–Outcome framework guided the controlled terminology and Medical Subject Headings (MeSH) keywords for the search strategy [14]. Population: dentists, dental specialists, dental hygienists and dental assistants; Exposure: noise levels and frequency; Comparison: no or a lower noise level; and Outcome: NIHL.

Eligibility criteria included (i) observational studies (cohort, case–control and cross-sectional); (ii) peer-reviewed journals; (iii) published in English and (iv) dentists, dental specialists, dental hygienists and dental assistants. Exclusion criteria included (i) case reports, case series, pilot studies, letters/editorials and opinion-based studies; literature reviews and qualitative surveys; experimental and *in vivo* studies; (ii) non-clinical dental personnel (dental technicians and dental receptionists); and (iii) papers published before 1990. Dental technicians were excluded as it is advised that they use protective devices for their ears [10], whereas clinicians do not since they need to communicate with patients.

A search was undertaken in March 2022 in PubMed, Scopus, Embase, Cochrane, Science Direct, Google Scholar and ProQuest. The search was repeated in March 2023 to ensure the inclusion of any newly published articles. A manual search of bibliographies of the included articles was conducted to identify other relevant articles. Table 1 presents the search strategy. Independent screening of titles and abstracts was done by five reviewers (A.C., E.M., G.T., J.H. and OM). Discrepancies and disagreements were resolved through consensus. A Cohen’s kappa value of 0.85 was set as the level of agreement [15]. Full-text analyses were performed independently by four reviewers (E.M., J.H., O.M. and G.T.) following the removal of duplicates. A record was kept for the reasons for exclusion.

**Table 1.** Search strategy

#	Search terms
#1	dent* OR ‘dental hygienist’ OR ‘dental assistant’ OR ‘oral health therapist’
#2	‘hearing impair*’ OR ‘hearing loss’ OR ‘hearing issues’ OR deafness OR ‘partial hearing’ OR ‘deaf*’ OR tinnitus OR ‘noise induced hearing loss’ OR ‘noise-induced hearing loss’ OR NIHL OR ringing OR ‘occupational hearing loss’ OR ‘transitory hypoacusis’
#3	‘ultrasonic scaler’ OR ‘ultrasonic instrument’ OR ‘ultrasonic equipment’ OR ‘ultrasonic cleaner’
#4	‘dent* handpiece’ OR ‘dent* drill’ OR ‘dent* air turbine handpiece’ OR ‘air turbine handpiece’
#5	‘dent* technician equipment’ OR ‘dent* technician instruments’ OR ‘dent* laboratory equipment’
#6	‘high volume suction’ OR ‘high volume evacuator’ OR ‘dental suction’ OR suction
#7	risk* OR impact OR effect OR influence OR hazard
#8	#1 AND #2 AND #3 AND #4 AND #5 AND #6 AND #7

The Newcastle–Ottawa Scale (NOS) was used for quality assessment of cohort and case–control studies [16], and a modified version for cross-sectional studies [17]. NOS evaluates selection, exposure and comparability with a maximum score of 9 for cohort and case–control studies, and a maximum score of 10 for cross-sectional studies. A score equal to or less than 5 indicates a high risk of bias, 6–7 a medium risk, and 8 or higher a low risk of bias. Two reviewers independently rated and compared the scores for each study (O.M. and G.T.). A third reviewer (A.C.) resolved any disagreements.

Two reviewers (J.H. and E.R.) extracted the data independently according to the *Cochrane Handbook for Systematic Reviews* [12]. This included study type and details (author, year, and publication and country), sample size, age of participants, statistical analysis, audiometric tests used and the direction of association of the investigated variables. Accuracy of information was checked by two reviewers (J.H. and E.R.). Data extraction was repeated to resolve any discrepancies.

Due to the heterogeneity of the statistical analysis and reporting of results of the included papers, presentation of results for the type of oral health professional and demographic/environmental variables associated with hearing impairment was either indicated as direct (+) or none (0). The number of studies with these respective associations, divided by the number of studies reporting on the respective variable, was expressed as a percentage. This was only calculated if four or more studies reported on a specific variable.

## Results

**Figure 1.**
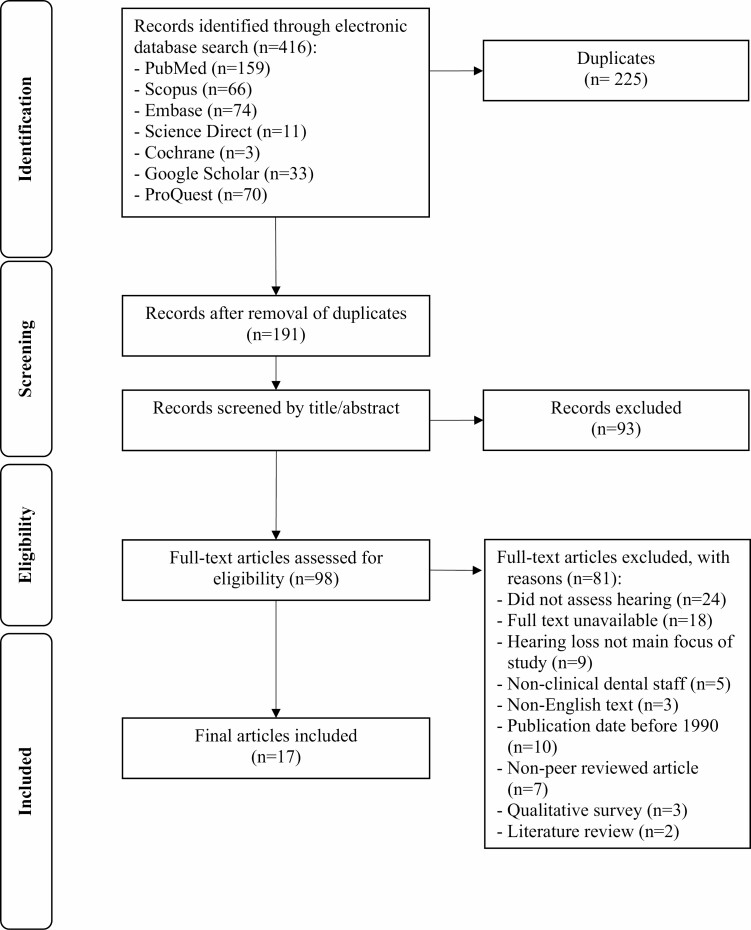
PRISMA flowchart.

Figure 1 shows the PRISMA flowchart for the selection of studies. The initial search identified 416 records. After the removal of duplicates and screening by title/abstract, 98 records remained for full-text review. This led to another 81 records being excluded with reasons, resulting in 17 records eligible for review and data extraction.

All variables in this systematic review were strictly quantitative, with the inclusion of questionnaire data only if it was subjected to statistical analysis to eliminate subjective perceptions of hearing loss. Supplementary Table 1 presents the characteristics of the included studies. One was a cohort (6%), 6 were case–control (35%) and the remaining 10 were cross-sectional studies (59%), conducted in 13 different countries. Participants’ hearing was measured through either a single or combination of audiometric tests, predominantly as pure tone audiometry, acoustic impedance, otoacoustic emission, distortion product otoacoustic emission and/or tympanometry. The statistical significance of the association between hearing loss and oral health professionals, demographic and environmental variables, was determined by conducting various bivariate or multivariate analyses. Most studies included dentists, dental specialists and/or dental assistants. Dental hygienists were included in one [18], and dental students in two studies [19,20]. Results for dental students were excluded as learning environments tend to have higher volumes of noise due to a large volume of students operating concurrently in large clinics [21,22]. The majority of studies found statistically significant hearing loss at high frequencies between 3 and 6 kHz [23–26].

Table 2 presents the quality assessment of the included studies. All studies had either a low (*n* = 14) or medium (*n* = 3) risk of bias. Of the 10 cross-sectional studies, 8 [20,23,26–31] had a low, and the remaining 2 [18,25] had a medium risk of bias, both of which did not mention blind testing. Only three studies did not account for confounding variables [11,25,27].

**Table 2.** Quality assessment of included studies

Author	Selection				Comparability		Outcome			Total score (Max: 9) (risk)
	Is the case definition adequate?	Representiveness of the cases	Selection of controls	Definition of controls	The subjects in different outcome groups are comparable, based on the study design or analysis	Confounding factors controlled	Ascertainment of exposure	Same method of ascertainment for cases and control	Non-response rate	
Cohort studies (*n* = 1)										
Gijbels et al. [33]	1	0	1	1	1	1	1	1	1	8 (low)
Case–control studies (*n* = 6)										
Ahmed et al. [32].	1	1	1	1	1	1	1	1	0	8 (low)
Al‐Omoush et al. [19]	1	1	1	1	1	1	1	1	1	9 (low)
Dierickx et al. [11]	1	1	1	1	1	0	1	1	0	7 (medium)
Gonçalves et al. [34]	1	1	1	1	1	1	1	1	0	8 (low)
Gurbuz et al. [24]	1	1	0	1	1	1	1	1	0	7 (medium)
Khaimook et al. [35]	1	1	1	1	1	1	1	1	0	8 (low)
Author	Selection				Comparability		Outcome		Total score (Max. 10) (risk)	
	Representative-ness of sample	Sample size	Non-respondents	Ascertainment of exposure	The subjects in different outcome groups are comparable, based on the study design or analysis.	Confounding factors controlled	Assessment of outcome	Statistical test		
Cross-sectional studies (*n* = 10)										
Alabdulwahhab et al. [27]	1	1	0	2	1	0	2	1	8 (low)	
Al-Rawi et al. [23]	1	1	0	2	1	1	1	1	8 (low)	
Chopra et al. [28]	1	1	1	2	1	1	2	1	10 (low)	
Daud et al. [29]	1	1	0	2	1	1	1	1	8 (low)	
Gabrielle et al. [30]	1	1	0	2	1	1	2	0	8 (low)	
Lopes et al. [25]	1	1	1	2	1	0	0	1	7 (medium)	
Shetty et al. [31]	1	1	1	2	1	1	2	1	10 (low)	
Theodoroff and Folmer [20]	1	1	0	2	1	1	2	1	9 (low)	
Willershausen et al. [26].	1	1	0	2	1	1	2	1	9 (low)	
Wilson et al. [18]	1	1	0	2	1	1	0	1	7 (medium)	

Table 3 presents the percentage of studies with either a direct (+) or no (0) association of the variable with NIHL included in this review. This was calculated from the results presented in Supplementary Table 1. Nine of the 11 studies (82%) found a direct association with hearing loss for dentists [20,24–26,28,32–35]. All five studies including dental specialists also indicated a direct association with hearing loss [23,25,27,30,31]. Two out of four studies (50%) reported a direct association between dental assistants and hearing loss [19,25].

**Table 3.** The association of type of oral health professional and demographic/environmental variables with NIHL

	Studies assessing the risk variable^a^ (*n*)	Direct association with hearing loss, *n* (%)	No association with hearing loss, *n* (%)
Type of oral health professional			
Dentist	11	9 (82)	2 (18)
Dental specialists	5	5 (100)	0 (0)
Dental assistants	4	2 (50)	2 (50)
Demographic and environmental variables			
Difference between ears	14	10 (71)	4 (29)
Years of clinical experience	11	9 (82)	2 (18)
Age	8	6 (75)	2 (25)
Gender	5	1 (20) (males)	4 (80)
Hours of exposure per day	4	1 (25)	3 (75)

^a^Calculations were only performed if four or more of the included studies (*n* = 17) reported on the variable.

The difference between the left and right ears was investigated in 14 studies, 10 of which (71%) reported a difference in hearing loss [19,20,23,25,27,28,31–34]. Years of clinical experience were reported in 11 studies. Nine (82%) found a direct association with hearing loss [11,20,23,24,30,31,33–35]. For gender, only one of five studies (20%) found a direct association with hearing loss reported for males [23]. Three of four studies (75%) found no association for hours of exposure per day [30,31,35].

## Discussion

Current literature shows no consensus on whether dental professionals are at risk of NIHL. The aim of this systematic review was to ascertain whether dental professionals, including dentists, dental specialists, dental hygienists and dental assistants, are at risk of hearing loss due to noise exposure in their work environment. A direct association with hearing loss in dentists and dental specialists was found in this review, combined with a difference between the left and right ears. Years of clinical experience were the most important demographic/environmental variable.

A strength of this review was the inclusion of observational studies with objective audiometric testing only, with pure tone audiometry reported in all included studies. As a result, all included studies had either a low (82%) or medium (18%) risk of bias. Further strengths were that similar results were reported from 13 different countries across the 17 included papers. Selection bias and associated publication bias were minimized by searching seven different databases, as well as implementing a duplicate screening strategy by independent reviewers.

A limitation of this review is that the majority of includes studies (59%) were cross-sectional, which in general does not provide good evidence since there is no temporal association. Heterogeneity of the statistical analysis and reporting of results of the included papers prevented a meta-analysis. Further limitations are that three studies did not account for confounding variables, and no studies reported on the impact of hearing loss on dental professionals’ quality of life. Although five studies reported on hearing loss in dental specialists, they were included in the study population with dentists [23,27,30]. Two studies specified the specialities included [25,31]. Another limitation was that only one of these drew comparisons, with one test indicating inner ear dysfunction in paediatric specialists, most likely due to cumulative noises of dental equipment and cries of babies and children [31]. A qualitative study concluded that prosthodontists were more significantly associated with hearing loss when compared to other specialities such as periodontology, dental surgery, paedodontics and orthodontics due to the regular use of equipment emitting louder noise, placing them at a higher risk of hearing loss when compared to other specialists [36].

The finding from this review is that significant hearing loss occurs at high frequencies between 3 and 6 kHz [23–26], especially since background noise of frequencies above 3 kHz plays a crucial role in localization and comprehension of speech [37].

Whereas clear associations with noise and hearing loss were found for dentists and dental specialists, this was inconclusive for dental assistants. A possible reason is that the average noise exposure for assistants was 6.6 h [29], which is less than the 8-h limit recommended by OSHA [3].

The majority of studies reported that the left ear was more affected by hearing loss in both dentists and specialists [23,27,28,32,33], most likely due to the left ear being closer to the noise-inducing equipment held by right-handed clinicians [33]. The left ear was also found to be more affected in dental assistants since it was closer to high-volume excavators and other equipment [19]. In contrast, two studies reported increased hearing loss in the right ear for both dentists and dental assistants [25,34], whereas equal hearing loss was reported in both ears in another study [20].

NIHL occurs with years of clinical experience. There is consensus in the literature that NIHL becomes apparent from 10 years or more of clinical experience [23,26,31,33,34]. Similarly, a significant correlation was found between sensorineural hearing loss and a work tenure of 15 years [35].

Age showed a positive association with NIHL in six out of eight studies (75%) [18,22,25,29,30,34], especially after the age of 40 [19,20,23,30,31,33–35]. Since presbycusis usually occurs from the age of 50 [30,31,35], results from this review for age should be considered in light of presbycusis as a confounding variable.

Hours of exposure per day showed no association with hearing loss. This variable is complex as the results are reliant on self-reported estimates due to the difficulty of measuring the exact duration of exposure [38]. Only one study found an association between the duration of exposure and hearing loss, where exposure to intensive noise-producing devices was significant at an average of 4 h per day [19]. The NIDCD advises the louder the noise, the shorter amount of time it takes to induce hearing loss [2]. This contrasts another recommendation which suggests exposure to noise levels over 85 dBA for 8 h is responsible for NIHL [9,36].

Future research should investigate the association between NIHL and dental specialists in more detail, the viability of hearing protection targeted towards the ear most at risk, noise levels produced by specific dental equipment, and comparisons between new/well-maintained versus old and poorly maintained dental equipment.

Conclusions from this review highlight the need for prevention and appropriate ear-protective devices to mitigate the implications of NIHL in the dental workplace [39]. Digital noise excluding headphones that excludes environmental noise, but allows the passage of the human voice, should be considered for the dental environment.

## Supplementary Material

kqad084_suppl_Supplementary_Table_1
