# Supplementary material for: Systematic review of hearing loss in dental professionals
Source: Occup Med (Lond). 2023 Sep 8;73(7):391–7. doi: 10.1093/occmed/kqad084 (PMC10588782; doi:10.1093/occmed/kqad084)
Supplement: kqad084_suppl_Supplementary_Table_1 [file kqad084_suppl_supplementary_table_1.docx]

**Table 2.** Characteristics of included studies

| Author  Year  Country | Age of participants: Range and Mean (SD) | Sample size  (*n*) | Risk of bias | Statistical analysis | Audiometric test | Direction of association^a^ | |
| --- | --- | --- | --- | --- | --- | --- | --- |
|  |  |  |  |  |  | Oral health professional | Demographic and environmental variables |
| Cohort studies (n=1) | | | | | | | |
| Gijbels et al. [33]  2006  Belgium | 28 (at second test) | 13 | Low | Wilcoxon matched-pairs tests | Pure tone audiometry | Dentists (+) | Difference between ears (+)  Years of clinical experience (+) |
| Case control studies (n=6) | | | | | | | |
| Ahmed et al. [32]  2013  India | Mean: 37 | 40 | Low | One-way ANCOVA (analysis of covariance) | Pure tone audiometry | Dentists (+) | Difference between ears (+)  Age (0) |
| Al‐Omoush et al. [19]  2020  Jordan | Range: 22-42  Mean: 30 | 306 | Low | Three-factor ANOVA  Paired samples t test  Pearson’s correlation | Pure tone audiometry  Tympanometry | Dentists (0)  Dental assistants (+)  Final year dental students (0)  Third year dental students (0) | Difference between ears (+)  Years of clinical experience (0)  Age (+)  Hours of exposure per day (+) |
| Dierickx et al. [11]  2021  Switzerland | Mean: 47 (SD 10.5), for dentists with >5 years’ experience  Mean: 26 (SD 3.1), for dentists with ≤5 years’ experience | 153 | Medium | One-way analysis of variance (ANOVA)  Bonferroni correction  Chi-square statistics | Pure tone audiometry | Dentists (0) | Years of clinical experience (+) |
| Gonçalves et al. [34]  2015  Brazil | Range:  23-61  Mean: 41 (SD 9.9) | 80 | Low | Descriptive statistics (mean, SD)  Student t test | Pure tone audiometry | Dentists (+) | Difference between ears (+)  Years of clinical experience (+) |
| Gurbuz et al. [24]  2013  Turkey | Mean: 48 (SD 6.3), for males  Mean: 46 (SD 7), for females | 80 | Low | Descriptive statistics (mean and SD) and P values (test not specified) | Pure tone audiometry | Dentists (+) | Difference between ears (0)  Years of clinical experience (+)  Gender (0)  Loudness of compressor (+)  Loudness of aerator/drill (+)  Distance of compressor to work area (-)  Distance of aerator to dentist’s ear (0) |
| Khaimook et al. [35]  2014  Thailand | Range: 25-50  Mean: 39 | 152 | Low | Descriptive study  Univariate analysis  Fisher’s exact test | Pure tone audiometry | Dentists (+)  Dental assistants (0) | Years of clinical experience (+)  Age (+)  Hours of exposure per day (0)  Speciality clinics (0) |
| Cross-sectional studies (n=10) | | | | | | | |
| Alabdulwahhab et al. [27]  2016  Saudi Arabia | Range: 25-40 | 76 | Low | Wilcoxon test | Pure tone audiometry  Distortion product otoacoustic emissions test | Dentists with mixed specialities (+) | Difference between ears (+) |
| Al-Rawi et al. [23]  2019  UAE | Range: 25-55 | 90 | Low | Discriminant analysis  Paired t test | Pure tone audiometry | Dentists with mixed specialities (+) | Difference between ears (+)  Years of clinical experience (+)  Age (+)  Gender (+ for males) |
| Chopra et al. [28]  2016  India | Range: 20-30 | 60 | Low | Paired t-test | Pure tone audiometry  Otoacoustic emission test | Dentists (+) | Difference between ears (+)  Pre- and post-exposure to ultrasonic scaling (+) |
| Daud et al. [29]  2011  Malaysia | Range: 28-54  Mean: 39 (SD 7.5) | 65 | Low | Descriptive statistics (mean, SD) | Pure tone audiometry | Dental assistants (0) | Difference between ears (0)  Years of clinical experience (0) |
| Gabrielle et al. [30]  2020  Indonesia | Range: 25-60  Mean: 36 | 69 | Low | Descriptive statistics (total, %) | Pure tone audiometry  Distortion product otoacoustic emissions test | Dentists with mixed specialities (+) | Years of clinical experience (+)  Age (+)  Gender (0)  Hours of exposure per day (0)  Days of exposure per week (0) |
| Lopes et al. [25]  2012  Brazil | Range: 23-59  Mean: 35 | 108 | Medium | Kruskall-Wallis test  Dunn test | Pure tone audiometry  Acoustic impedance test | Dentists (+)  Prosthodontists (+)  Dental assistants (+) | Difference between ears (+) |
| Shetty et al. [31]  2020  India | Range: 23-55  Mean: 35 (SD 7.9) | 60 | Low | Two-way analysis of variance  Tukey HSD test, as a post hoc test | Pure tone audiometry  Otoacoustic emission test | Prosthodontist (+)  Endodontist (+)  Periodontist (+)  Paedodontist (+) | Difference between ears (+)  Years of clinical experience (+)  Age (+)  Gender (0)  Hours of exposure per day (0) |
| Theodoroff & Folmer [20]  2015  USA | Mean (dentists): 54  Mean (dental students): 29 | 37 | Low | Descriptive statistics  2-tailed t test  One-way ANOVA with Bonferroni correction  Post hoc | Pure tone audiometry | Dentists who routinely use high-speed handpieces (+)  Dentists who do not routinely use high-speed handpieces (0)  Dental students (0) | Difference between ears (+)  Years of clinical experience (+)  Age (0) |
| Willershausen et al. [26]  2014  Germany | Range: 34-74  Mean: 52 (SD 9.6) | 108 | Low | Mann-Whitney U test | Pure tone audiometry | Dentists (+) | Difference between ears (0)  Age (+)  Gender (0)  Living in densely populated urban setting (+) |
| Wilson et al. **[18]**  2002  USA | Mean: 43 | 40 | Medium | Analysis of variance (ANOVA) | Pure tone audiometry | Dental hygienists (+) | Difference between ears (0)  High vs low ultrasonic usage (+) |
